# Case report: Efficacy of immune checkpoint inhibitors for high tumour mutational burden malignant phyllodes tumours of the breast as revealed by comprehensive genomic profiling

**DOI:** 10.3389/fimmu.2025.1549452

**Published:** 2025-02-24

**Authors:** Hiroo Katsuya, Haruhiko Sano, Haruna Sano, Tatsuya Mihashi, Chiho Nakashima, Keita Kai, Shinya Kimura

**Affiliations:** ^1^ Division of Haematology, Respiratory Medicine and Oncology, Department of Internal Medicine, Faculty of Medicine, Saga University, Saga, Japan; ^2^ Department of Pathology, Saga University, Saga, Japan

**Keywords:** immune checkpoint inhibitor (ICI), tumour mutational burden (TMB), malignant phyllodes tumor of the breast, comprehensive genomic profiling (CGP), microsatellite instability

## Abstract

High tumour mutational burden (TMB-high), identified through comprehensive genomic profiling (CGP), is a biomarker that predicts the efficacy of immune checkpoint inhibitors. CGP testing is recommended for rare cancers with limited effective treatment options. Here, we provide the first report of a malignant phyllodes tumour of the breast demonstrating TMB-high status and effective treatment with pembrolizumab. The patient initially underwent right breast tumour resection and was diagnosed with a malignant phyllodes tumour. Recurrence was observed at the same site with metastases to the pulmonary hilar and right axillary lymph nodes. CGP testing revealed TMB-high status and microsatellite stable. Pembrolizumab was initiated after chemotherapy for the soft tissue sarcoma. After two treatment cycles, imaging revealed a partial response of the pulmonary hilar lymph nodes and necrotic enlargement of the right axillary lymph node. After four cycles, all lymph nodes had reduced in size. Eight months after ten cycles of pembrolizumab treatment, multiple new nodules were observed in both lungs, indicating disease progression. This case highlights the utility of CGP testing in rare cancers, demonstrating the first evidence of TMB-high malignant phyllodes tumours of the breast responding to pembrolizumab and underscoring the value of expanding CGP testing to improve therapeutic strategies for rare cancers.

## Introduction

Immune checkpoint inhibitors (ICI) have significantly improved the therapeutic outcomes of advanced malignant tumours, irrespective of cancer type. Two well-known indicators of ICI effectiveness are high tumour mutational burden (TMB-high; >10 mutations per megabase, mut/Mb) (1, 2) and high microsatellite instability (MSI-high) (3, 4). These indicators serve as valuable cross-sectional metrics regardless of the cancer type. TMB, defined as the total number of somatic mutations per coding area of a tumour genome, is a measure of all nonsynonymous coding mutations in the tumour exome (5). Because highly mutated tumours can produce many neoantigens, TMB-high has been hypothesised to be associated with an improved response to treatment with immune checkpoint blockades (6, 7).

Phyllodes tumours of the breast are rare neoplasms that account for less than one percent of all breast tumours (8, 9). Histologically, phyllodes tumours are classified as benign, borderline, or malignant based on the assessment of four features: degree of stromal cellularity and atypia, mitotic count, stromal overgrowth, and the nature of the tumour borders (10). Surgical excision is the primary choice when the tumour is resectable, while chemotherapy is employed in cases of metastatic disease using treatment guidelines for soft tissue sarcomas (11). However, most of these regimens have limited or short-lived benefits. For these rare cancers, comprehensive genomic profiling (CGP) testing has the potential to expand the treatment options (12, 13). Here, we present the first documentation of a case demonstrating the efficacy of the immune checkpoint inhibitor, pembrolizumab, in a patient with malignant phyllodes tumours exhibiting TMB-high.

## Case report

**Figure 1:**
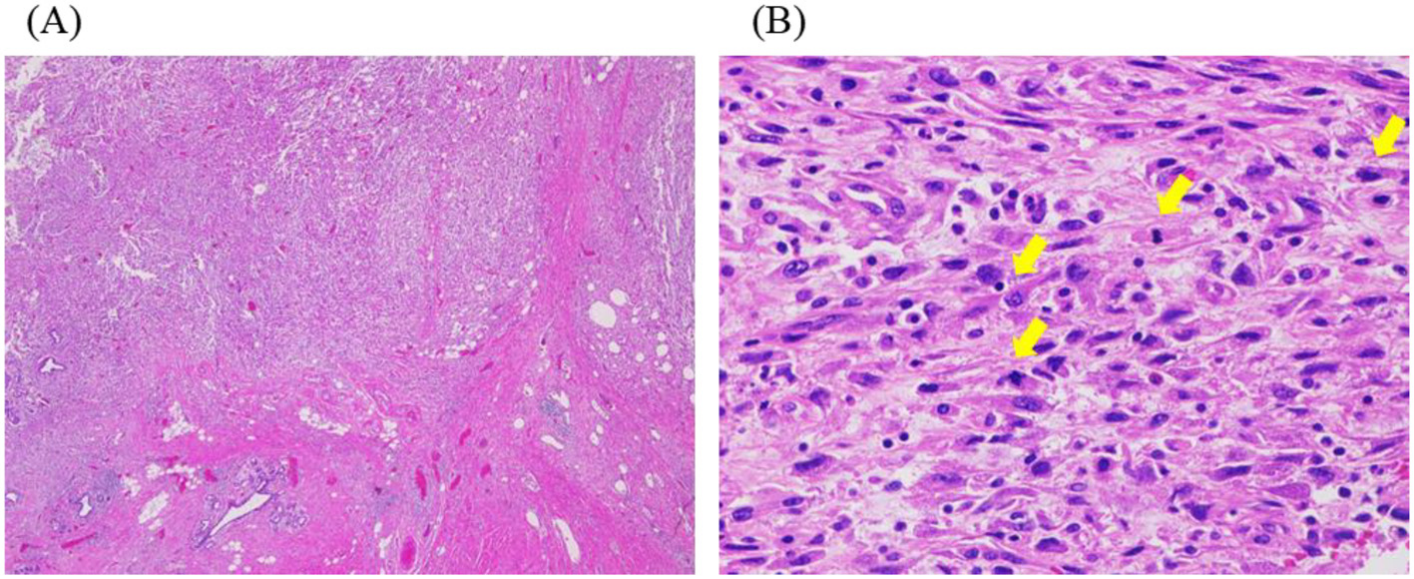
Histopathological findings of primary tumour. **(A, B)** Haematoxylin-eosin staining. **(A)** Spindle-shaped tumour cells are invasively proliferating (original magnification: ×20). **(B)** The spindle-shaped tumour cells showed distinct cytological atypia, and many mitotic figures, indicated by the yellow arrows, are observed (original magnification: ×400).

**Figure 2:**
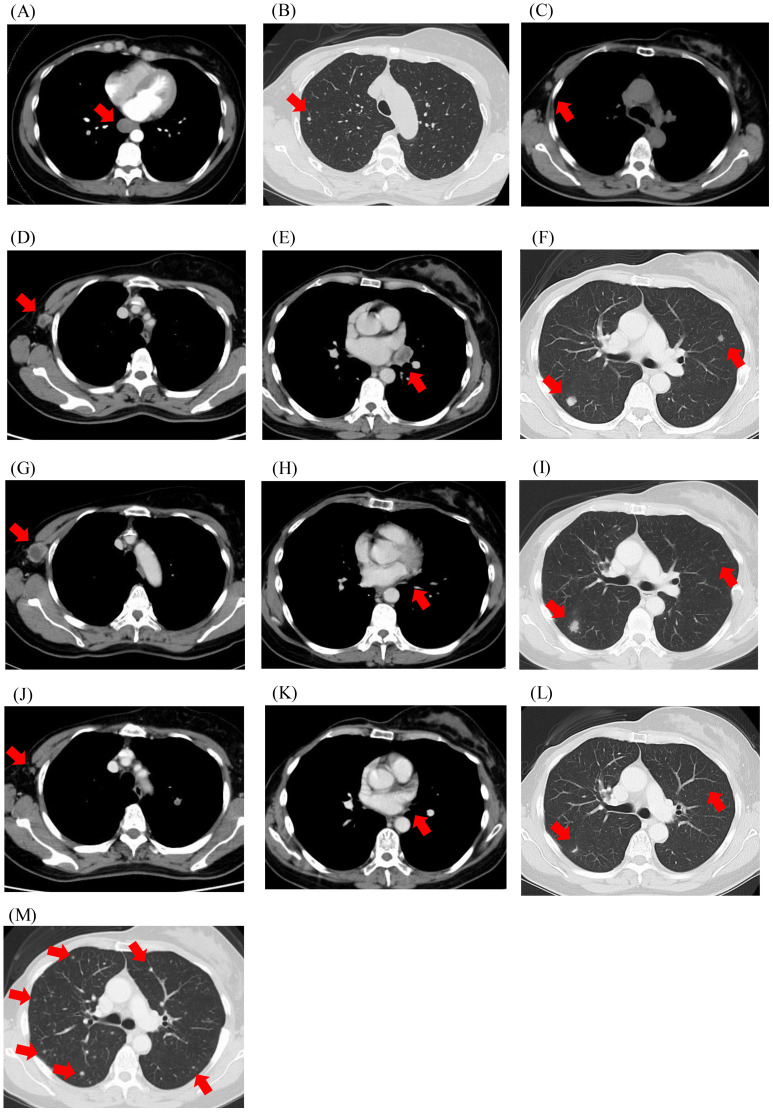
Computed tomography imaging. **(A)** Upon diagnosis of distant metastasis; **(B, C)** Before the initiation of chemotherapy; **(D-F)** Before the initiation of pembrolizumab; **(G-I)** After two cycles of pembrolizumab; **(J-L)** After four cycles of pembrolizumab; **(M)** At disease progression after 10 cycles of pembrolizumab. Red arrows indicate metastatic lesions.

A 48-year-old woman noticed a thumb-sized lump in her right breast and underwent tumour resection, which was diagnosed as a malignant phyllodes tumour (**Figure 1**). The patient is a known hypertation with no history of other cancers. There was no family history of cancer either. Four months later, the tumour recurred in the same location, and the patient underwent a right mastectomy, resulting in a pathological diagnosis of a recurrent malignant phyllodes tumour. Four months after the mastectomy, computed tomography (CT) revealed a mass in the right lung (**Figure 2A**), and she underwent partial resection of the right lower lobe of the lung via thoracoscopy. The mass was pathologically diagnosed as a metastasis of the malignant phyllodes tumour. Subsequent CT scans showed metastases in both lung fields and the right axillary lymph nodes (**Figures 2B, C**), prompting her visit to our facility.

Based on the treatment protocol for soft tissue sarcoma, doxorubicin, pazopanib, and eribulin were used sequentially, with the best objective response being stable disease. The progression-free survivals for doxorubicin, pazopanib, and eribulin were 2.9, 2.2, and 5.1 months, respectively. During eribulin treatment, a resected metastatic lung specimen was subjected to the CGP test (FoundationOne CDx). Biomarker findings indicated TMB-high with 37 mut/Mb; however, MSI could not be determined. Genomic analysis revealed variants of *PIK3R1*, *MSH2*, and *TERT;* the *MSH2* and *TERT* genes had low variant allele frequencies and were not considered hereditary. Subsequent verification using multiplex PCR fragment analysis confirmed the tumour cells were microsatellite stable (MSS).

Based on the TMB-high status, pembrolizumab treatment (200 mg every three weeks) was initiated (**Figures 2D–F**). Two weeks after starting treatment, the patient developed fever at 38°C and experienced fatigue. However, neither blood tests nor imaging studies revealed signs of infection, nor were there any findings suggestive of organ dysfunction related to ICI-associated adverse events. The fever was thus attributed to grade 1 ICI-related effects and oral prednisolone (20 mg/day) was initiated. The fever and generalised fatigue improved, and pembrolizumab treatment was continued with gradual tapering of prednisolone.

CT imaging after two cycles of pembrolizumab revealed a partial response in the hilar lymph node lesions, enlargement of the right axillary lymph nodes with necrotic changes, and no significant changes in the pulmonary lesions (**Figures 2G–I**). After four cycles, the lymph nodes and pulmonary nodules showed significant shrinkage (**Figures 2J–L**). However, after ten cycles, 7.7 months after starting pembrolizumab, CT imaging showed the emergence of multiple nodular shadows in both lungs, leading to a diagnosis of progressive disease (**Figure 2M**). The treatment was switched to trabectedin, which achieved the best response for stable disease, and was continued for 8.3 months with 11 cycles. The patient died 2 years and 9 months after the detection of the lung metastases.

## Discussion

To date, three reports have summarised the results of using CGP for malignant phyllodes tumours of the breast (13–15). Nozad et al. reported no cases with TMB-high (13). Rosenberger et al. reported that the median TMB across samples was 2.5 mut/Mb (range 0-12 mut/Mb), and that only three cases (1.45%) were TMB-high (≥10 mut/Mb) (15). Data from our patient were included in a report by Suzuki et al., accounting for the only TMB-high case out of 60 specimens in Japan’s National Clinical Genomic Testing Registry (C-CAT database) (14). Due to the summarised nature of the CGP results provided in these reports, we are unable to assess the role of patient-specific factors, tumour features, therapeutic interventions, or patient outcomes. Thus, we provide the first detailed report of a patient with a malignant phyllodes tumour of the breast presenting with TMB-high, including the effect of immune checkpoint inhibitor administration.

TMB-high is most frequently found in melanoma, head and neck squamous cell carcinomas, urothelial carcinoma, and non-small and small cell lung cancers (1), whereas the proportion of soft tissue sarcomas that are TMB-high is extremely low (16). In rare cancers such as soft tissue sarcomas, the number of available treatment options is limited, and achieving sufficient therapeutic efficacy with the currently recommended chemotherapy regimen remains challenging. Particularly for these rare cancers, CGP testing offers the potential to expand treatment options.

The FoundationOne CDx assay, a commercially available CGP test, is a companion diagnostic test for pembrolizumab that detects TMB-high in patients with solid tumours. The efficacy of pembrolizumab was established in the KEYNOTE-158 single-arm phase II trial (2). In this trial, the objective response rate was 29 percent among patients with TMB-high tumours. Considering that the median survival time for patients with metastatic malignant phyllodes tumours of the breast has been reported to be 11–24 months (11, 17), pembrolizumab treatment tailored to the CGP results likely contributed to our patient’s 30-month survival.

MSI is characterised by genetic hypermutability that generates excessive amounts of short insertion/deletion mutations in the genome (3). It generally occurs in microsatellite DNA sequences and is associated with a deficiency in DNA mismatch repair (MMR) in tumours. Tumours that lack the MMR mechanism harbour more mutations than tumours of the same type without MMR defects. The CGP analysis for this patient resulted in “MSI could not be determined”. Subsequent verification using multiplex PCR fragment analysis confirmed that the tumour cells were MSS. Most tumours expressing MSI-high or deficient MMR also have high TMB levels; however, not all TMB-high tumours are MSI-high or MMR-deficient, as observed in our patient (18). In a phase II TAPUR basket study, the objective response to pembrolizumab was limited (11 percent) even in patients with TMB-high colorectal cancer, most of whom had proficient MMR and MSS (19). However, a partial response was observed in one patient with both TMB-H and a polymerase epsilon (*POLE*) gene mutation. *POLE* missense mutations have been reported to generate proofreading defects, resulting in TMB-high in proficient MMR tumours and the sensitisation of tumours to checkpoint blockade immunotherapy (20). There have been no reports of *POLE* gene abnormalities in soft tissue tumours, such as phyllodes tumours, which were not observed in our patient. In an analysis utilising multi-omics datasets from The Cancer Genome Atlas program, among the MSS subtype, TMB-high was observed in only 6% of colorectal cancers, 17% of oesophageal cancers, 2% of gastric cancers, and 10% of endometrial carcinomas (21). Compared with MSS patients with TMB-low in oesophageal and gastric adenocarcinoma, mutations in *TP53*, *APC*, *KRAS*, and *ERBB4* as well as amplifications of *ERBB2* were more prevalent in TMB-high and MSS patients (22). Further research and consolidation of cases are needed to investigate the genetic abnormalities associated with tumours that are TMB-high and proficient in MMR and MSS.

Here, we report a case of malignant phyllodes tumours of the breast in which a CGP test identified a TMB-high status responding to pembrolizumab therapy. This case underscores the utility of CGP testing for rare cancers and highlights the need for continued accumulation of evidence regarding CGP-directed outcomes in rare cancers.

## Data Availability

The original contributions presented in the study are included in the article. Further inquires can be directed to the corresponding author.
